# Incidental Category Learning and Cognitive Load in a Multisensory Environment Across Childhood

**DOI:** 10.1037/dev0000472

**Published:** 2018-01-08

**Authors:** H. J. Broadbent, T. Osborne, M. Rea, A. Peng, D. Mareschal, N. Z. Kirkham

**Affiliations:** 1 Centre for Brain and Cognitive Development, Birkbeck, University of London

**Keywords:** multisensory integration, cognitive development, incidental learning, cognitive load

## Abstract

Multisensory information has been shown to facilitate learning (Bahrick & Lickliter, 2000; Broadbent, White, Mareschal, & Kirkham, 2017; Jordan & Baker, 2011; Shams & Seitz, 2008). However, although research has examined the modulating effect of unisensory and multisensory distractors on multisensory processing, the extent to which a concurrent unisensory or multisensory cognitive load task would interfere with or support multisensory learning remains unclear. This study examined the role of concurrent task modality on incidental category learning in 6- to 10-year-olds. Participants were engaged in a multisensory learning task while also performing either a unisensory (visual or auditory only) or multisensory (audiovisual) concurrent task (CT). We found that engaging in an auditory CT led to poorer performance on incidental category learning compared with an audiovisual or visual CT, across groups. In 6-year-olds, category test performance was at chance in the auditory-only CT condition, suggesting auditory concurrent tasks may interfere with learning in younger children, but the addition of visual information may serve to focus attention. These findings provide novel insight into the use of multisensory concurrent information on incidental learning. Implications for the deployment of multisensory learning tasks within education across development and developmental changes in modality dominance and ability to switch flexibly across modalities are discussed.

Successful integration of information from multiple sensory systems is imperative to constructing meaningful representations of the environment. This is particularly salient in formal learning environments, which typically demand attention from stimuli to different modalities. The ability to discriminate between relevant and extraneous environmental information is critical, particularly as learning tasks do not ordinarily occur in isolation. Developmental changes during the primary school years in ability to suppress irrelevant background information (Osman & Sullivan, 2014; Ridderinkhof, van der Molen, Band, & Bashore, 1997) have important implications for learning. However, age-related changes in ability to attend to and learn information on a primary task, while engaged in a concurrent unisensory or multisensory task has scarcely been examined.

The presentation of redundant multisensory cues can facilitate learning in adults (Fifer, Barutchu, Shivdasani, & Crewther, 2013; Lehmann & Murray, 2005; Seitz, Kim, & Shams, 2006; Shams & Seitz, 2008) and modulate attention in infants (Bahrick, Flom, & Lickliter, 2002; Bahrick & Lickliter, 2000; Bahrick, Lickliter, & Flom, 2004; Gogate & Bahrick, 1998; Lewkowicz, 2000; Lewkowicz & Kraebel, 2004; Richardson & Kirkham, 2004). These findings suggest that amodal properties presented unimodally are less salient than when experienced redundantly across two senses. However, despite our understanding of the development of multisensory integration, the efficacy of multisensory learning tools during childhood remains relatively unaddressed. In addition, multisensory cues in educational tools may only moderately resemble the stimuli used to address cross-modal sensitivity in laboratory studies.

In naturalistic learning tasks, the beneficial effects of complementary or redundant multisensory compared with unisensory information continue to mature between 5 and 10 years of age (Broadbent et al., 2017; Jordan & Baker, 2011). However, in the presence of competing unisensory and multisensory distractors, facilitation effects of multisensory information are diminished both in children and adults (Downing, Barutchu, & Crewther, 2015). Similarly, the processing of multisensory information is modulated in the presence of background auditory noise, as evidenced particularly by slower reaction times (RTs) to targets (Steenken, Colonius, Diederich, & Rach, 2008).

When unimodal visual stimulation is extraneous to the current task, such as in a vibrant classroom display, attention allocation is affected leading to poorer learning outcomes in 5-year-olds, compared with learning in sparse classroom environments (Fisher, Godwin, & Seltman, 2014). Likewise, in selective attention tasks, young children have more difficulty than older children and adults in avoiding processing peripheral stimuli (Enns & Akhtar, 1989) and more difficulty entirely filtering out irrelevant stimuli (Ridderinkhof et al., 1997). Also, 7-year-olds are more distracted by peripherally presented letters than young adults (Huang-Pollock, Carr, & Nigg, 2002). These age-related improvements in maintaining focused attention and reduced distractibility are attributed to the protracted development of working memory and inhibitory control (Diamond, 2006).

Although informative as to the role of same-modality or cross-modal background distractors or “noise” on multisensory processing, such findings tell us little about the role of unisensory and multisensory information when pertaining to concurrent tasks that increase attentional load. Learning exercises often require an individual to allocate their attention appropriately between two or more parallel tasks. For example, in a classroom environment, children are often expected to engage in learning tasks while attending to further instructions from a teacher or whiteboard. Currently, little is understood regarding the effect of engaging in a concurrent unisensory or multisensory task on learning in the primary task. That is to say, would the presentation of multisensory concurrent task information support attention to one or both tasks, or encumber the cognitive system and therefore serve as a greater distraction than unimodal information? Furthermore, does the role of concurrent task stimuli in different modalities differ across development, particularly given changes in sensory dominance during childhood (Nava & Pavani, 2013)?

The limited nature of attentional resources as proposed by the perceptual load theory (Lavie, 1995, 2005; Lavie & Tsal, 1994) determines the extent to which stimuli external to the primary task are processed. Due to limited attentional capacity, when perceptual load is high (there is a large amount of information involved in the processing of task stimuli), distractors and peripheral stimuli are less likely to be processed. In contrast, in conditions of low load, all available stimuli are processed, including distractors (Lavie, 2005).

In adults, under conditions of high cognitive load, attentional resources have been shown to be shared across modalities. For instance, Macdonald and Lavie (2011) found that on a perceptually demanding visual task, conscious awareness of an auditory tone was reduced compared with a similar task with lower perceptual demands. Likewise, high visual load has been found to negatively affect recall for auditory information, further indicating a possible cross-modal perceptual load effect on memory (Murphy, Groeger, & Greene, 2016). Furthermore, such findings suggest that it is not just visual attention that is affected by visual load; the amount of input for a given sensory modality may be limited by attentional resources on supporting systems.

However, there remains a developmental question. That is, in young children for whom integration of bimodal information may not optimal (e.g., Barutchu, Crewther, & Crewther, 2009; Gori, Del Viva, Sandini, & Burr, 2008; Nardini, Bales, & Mareschal, 2015; Nardini, Jones, Bedford, & Braddick, 2008), and thus presentation of bimodal information not be processed as readily, would cognitive load be effected differently? On the one hand, if the presentation of cross-modal stimuli were to actually *increase* cognitive load, it can be inferred that concurrent multisensory information (that which is external to the primary task but still attended to) would result in a higher level of interference than unisensory information, as these tasks demand the engagement of attentional resources in two modalities. As a result, one hypothesis is that multisensory information on a concurrent task may interfere with learning to a greater extent in younger than in older children, by requiring heightened selective attention, a skill that undergoes protracted development. On the other hand, however, in accordance with the *intersensory redundancy hypothesis* (Bahrick et al., 2004), the cross-modal stimuli on a secondary task would provide redundant information, which could result in enhanced perception of the concurrent task stimuli. If this is the case, amodal cross-modal stimuli might be processed quickly without increasing cognitive load.

In one study, on a visual selective attention task, (Matusz et al., 2015) examined the role of perceptual load using peripheral unisensory and multisensory distractor stimuli. The extent to which an increase in visual perceptual load decreased the effects of peripheral distractors was dependent on both the modality of the distractor and the age of the participant. At low cognitive load, unimodal visual distractors had a greater effect on performance in younger children, whereas higher visual load on the central task eliminated distractor effects at all ages. When distractors were audiovisual (multisensory), however, adults showed distraction effects both at high and low cognitive load conditions. A different pattern of performance was seen in children. At low load, 6- and 11-year-olds were significantly distracted by multisensory information, whereas a higher visual load decreased distraction effects of multisensory items, particularly in the youngest group. These findings go some way to demonstrating that processing of multisensory stimuli is more flexible than the literature on redundancy suggests, and highlights the importance of examining the differential effects of multimodal information on learning with development.

It is also currently unknown whether changes in sensory modality dominance across development, from auditory dominance in infants and children aged 4 to 6 years of age to visual dominance in adults (Colavita, 1974; Nava & Pavani, 2013; Robinson & Sloutsky, 2004), would influence the role of concurrent task modality on multisensory learning. According to the auditory dominance account, where visual and auditory information compete for attentional resources in children, greater attentional resources are delegated to auditory stimuli (given their dynamic and transient nature) at a cost to visual processing (Robinson & Sloutsky, 2010a, 2010b; Sloutsky & Napolitano, 2003); although the auditory processing is not invulnerable to interference from the concurrent visual information (Thomas, Nardini, & Mareschal, 2017). In light of this, an alternative hypothesis for the current study is that age-related differences in multisensory processing of information on two concurrent tasks may be modulated by a transition from auditory to visual modality dominance. In particular, concurrent auditory information may result in greater levels of distraction than visual in younger children, with a change to visual dominance with age.

In line with our previous research examining the role of multisensory information on learning across childhood, an incidental category learning task was used in the current study. In naturalistic environments, learning does not always arise from explicit instruction. Indeed, classroom-based teaching predominantly includes incidental learning objectives. For example, on a mathematical learning task that involves counting different objects, the learning of concepts such as categorical information or other perceptual properties of the items may be incidental to the initial task, but are important comprehensively. For this reason, it is important to examine the extent to which unisensory and multisensory concurrent response tasks interfere with or support incidental learning across childhood.

The aim of the present study, therefore, was to investigate the extent to which a concurrent unisensory or multisensory cognitive load task would interfere with or support incidental learning of multisensory categories across the primary school years. Consistent with previous findings, it was predicted that performance would improve with age on both a primary learning task and a concurrent task, but that incidental category learning would be differentially affected across development depending on the modality of a concurrent task in which the individual is engaged. In line with typical developmental changes, we predicted age-related improvements in incidental learning, and differential effects of concurrent task condition on learning across all groups. In particular, in concurrent tasks comprising multisensory (i.e., audiovisual) stimuli, it was predicted that incidental category learning in a primary task would be modulated to a different extent than with unimodal concurrent tasks, but that this would be differentially affected between 6 and 10 years of age.

## Method

### Participants

Data from 180 children were included in the study. Participants were selected from three separate school years (1, 3, and 5; *N* = 60 per group). This resulted in three age groups as follows: 6-year-olds, mean (*SD*) age = 6.21 (0.32) years, 32 males; 8-year-olds, mean (*SD*) age = 8.24 (0.39) years, 33 males; and 10-year-olds, mean (*SD*) age = 10.15 (0.45) years, 29 males. Participants in each group were randomly allocated to one of three concurrent-task (CT) conditions (visual [V], auditory [A], or audiovisual [AV]), in a between-subjects design (*N* = 20 per condition in each age group). Sample sizes for each group, per condition, were determined by power analysis for ANOVA with *df* = 1, f = 0.40. Children were recruited from local primary schools and informed written parental consent was obtained for each participant, in accordance with the Department of Psychological Sciences, Birkbeck University of London Ethics Committee, 131453, project title: “Learning from Multisensory Cues.” All participants had normal hearing and normal (or corrected-to-normal) vision, and no known developmental or neurological disorder, as assessed on the parental consent form. All testing was conducted in a quiet room within the participant’s school and children were rewarded for participating with a certificate and stickers. Testing sessions for each participant lasted approximately 20 min.

### Stimuli

The *frogs and stars* task is a modified version of the audiovisual condition of the Multisensory Attention Learning Task (MALT; Broadbent et al., 2017), with an integrated concurrent task (CT). Using a between-subjects design, the CT consisted of either a visual (V), auditory (A) or audiovisual (AV) task in which participants were asked to count the number of stars (V), dings (A), or dinging stars (AV) that occurred during the game, depending on the participant’s assigned condition. CT stimuli appeared quasi-randomly (hard-coded, with the same permutation for all participants) during interstimulus intervals (ISIs) on the MALT, for 500-ms durations (ISIs were 1,500/2,000 ms durations, so CT stimuli occurred for the first 500 ms of an ISI). Visual CT stimuli (a yellow star subtending an approximate 3° visual angle) appeared in the center of the screen (the same location as the target and nontarget images on MALT trials). Auditory CT stimuli were presented at 44kHz and around 70–75 dB through dual-channel closed-back headphones. For audiovisual CT stimuli (dinging stars), the visual (star), and auditory (ding) elements of the cues were presented concurrently with the same onset and offset. Task stimuli were presented using the Psychophysics Toolbox extension for MATLAB (Brainard, 1997), and presented on a 15 in. laptop screen approximately 50 cm in front of the participant.

In the MALT, all target (frog) and nontarget (cat, dog, elephant, goat, owl, pig) stimuli were presented with synchronous and complementary visual and auditory features. All visual images were forward facing depicting a head and body with (front) legs for consistency and to maintain a level of similarity across stimuli. Auditory stimuli consisted of congruent animal sounds, consistent with the different visual animal stimuli.

Two categories of target (frog) stimuli could be differentiated by visual features (few spots [2–3]) or many spots [7–8]) and auditory features (high and long-short “*rib*-bit” sound or a deep and short-long “rib-*bit*”). Spots varied in colors and sizes across category members (10 within-category members). Similarly, within-category auditory features varied in pitch at 0.5 semitone intervals (10 within-category members). Auditory stimuli were created from digital animal sound (.wav) files and manipulated using Audacity Digital Audio Editor Software into 300-ms sound files. For counterbalancing, two combinations of audiovisual categorizing features were used, that is, either Family 1: few spots with high ribbit and Family 2: many spots with deep ribbit, or, Family 1: few spots with deep ribbit and Family 2: many spots with high ribbit. Participants were not made aware of these categories.

### Procedure

As a measure of auditory working memory, each participant initially completed the Digit Span Backwards (DSB) task from the British Ability Scales–II (Elliott, Smith, & McCulloch, 1996). This measure of auditory working memory was primarily included as a proxy for cognitive ability to assess whether age groups could be considered as performing as expected for age, in line with other previous studies (e.g., Broadbent et al., 2017).

#### Stimuli familiarization

Before presentation of the *frogs and stars* task, participants were first familiarized to the MALT stimuli to check that they were able to see and hear the exemplar of each animal in the task. Participants were shown one of each animal in turn and asked whether they were able to hear and see the exemplar. All participants answered affirmatively for each of the seven exemplars and so continued with the task.

#### Frogs and stars task

Participants were instructed to press the space bar as quickly as possible whenever a frog (target animal) appeared (visually on the screen and auditorily through the headphones) while ignoring (inhibiting a response) to any other animal stimuli. Participants were not made aware that there would be two different families (categories) of target stimuli nor asked to learn their habitat locations. This was in order to examine *incidental* learning of these target categories.

The task consisted of 200 trials, separated into five blocks of 40 trials, with each block separated by a motivation screen to provide a rest break. Across the task, target stimuli (frogs) were presented on 40% of trials (80 trials; 40 exemplars from each family). Twenty of each nontarget stimuli were presented randomly throughout the task. Participants were also asked to count the number of “stars,” “dings,” or “dinging stars” throughout each block, depending on the CT condition to which they had been allocated (V, A, or AV, respectively). CT stimuli were presented during a number of interstimulus intervals (ISIs) following nontarget trials in each block.

**Figure 1 fig1:**
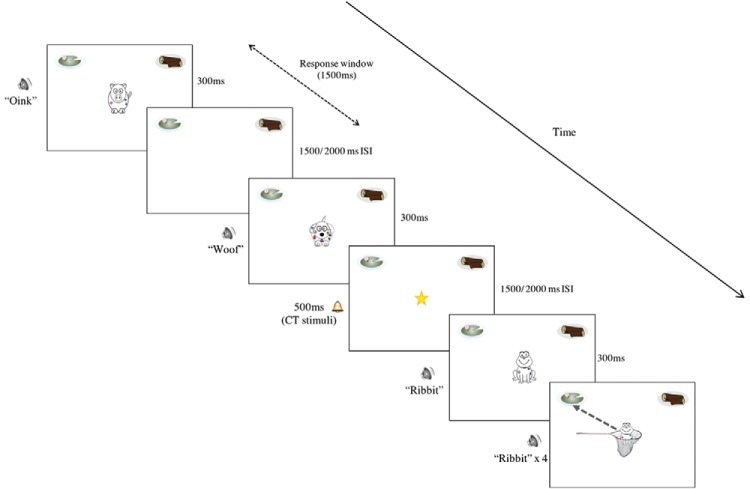
Task presentation order. Screen with star indicates bimodal concurrent task stimulus (AV). Final depicted screen appeared following a correct keypress response to target stimulus, with dashed arrow indicating direction of movement to correct category habitat. Adapted from *Incidental Learning in a Multisensory Environment Across Childhood*, by H. J. Broadbent, H. White, D. Mareschal, N. Z. Kirkham, 2017, *Developmental Science*.

The task screen consisted of a white screen with an image of a lily pad in the top left-hand corner and an image of a log in the top right-hand corner. On each trial, an animal image (target or nontarget) appeared individually in the center of the screen for 300 ms, simultaneously with the corresponding 300 ms auditory cue (animal sound). If the space bar was (correctly) pressed after the presentation of a target stimulus, the same frog reappeared within a “net” (see Figure 1, final screen). The frog then immediately traveled to the top left- or top right-hand corner of the screen to the correct frog habitat (e.g., frog exemplars from one family consistently traveled to the lily pad habitat, while frog exemplars from the other family traveled to the log habitat, counterbalanced across participants). Travel time to habitat was 2,000 ms. The corresponding 300-ms audio file for that frog was also played simultaneously three times until the frog reached the correct habitat. This was for consistency with exposure to the visual stimuli for incidental learning of categorical information. Following movement to the habitat, the target image was then paused for an additional 1,000 ms to avoid disorientation caused by an immediate appearance of the next stimulus. If the button was pressed incorrectly for a nontarget animal, no feedback was given and the task continued to the next trial after either a 1,500 ms or 2,000 ms ISI.[Fig-anchor fig1]

As mentioned above, during some of the ISIs (following nontarget MALT trials) in each block, a 500-ms CT stimulus was played. During Block 1, it was presented eight times, Block 2 = 12 times, Block 3 = nine times, Block 4 = 11 times, and Block 5 = 10 times (total = 50 CT stimuli). The CT stimuli, therefore, only appeared following 25% of the MALT trials. During rest breaks, participants were asked how many of each CT stimuli they had counted during that block. This was presented to participants both as a question on the screen and orally by the experimenter, to account for poorer reading and attentional skills in the youngest children. Another instruction screen was then displayed reminding them to start counting the CT stimuli again in the next block. As before, this instruction was also read aloud by the experimenter for all participants, to make sure that they remembered to begin counting from zero.

Across the whole task, target stimuli (frogs) were presented on 40% of trials (80 trials; 40 exemplars from each family). Twenty of each nontarget (distractor) stimuli were presented randomly throughout the task.

#### Category identification test

To examine the extent of incidental category learning on the *frogs and stars* task, participants were then asked to complete a category identification task. Eight audiovisual exemplars from each category (16 total) were presented in a random order. Participants responded to whether the frog had lived at the lily pad or the log during the game. Participants were presented with each frog exemplar individually, and no feedback was given throughout the identification task. Total correct categorization responses were recorded. Following the categorization test, a measure of explicit categorization knowledge was then given, where participants were asked “Can you tell me how you decided where each frog lived? What made them belong to each family?”

## Results

### Auditory Working Memory

Digit Span Backwards (DSB) raw ability scores were converted to standardized T-Scores and compared across groups using a one-way analysis of variance (ANOVA). No significant difference was found between groups; 6 years: mean (*SD*) = 55.97 (8.76); 8 years = 53.90 (9.75); 10 years = 53.37 (8.53), *F*(2, 179) = 1.39, *p = .252*, showing participants in each group were performing at a cognitive level expected for their age.

### Frogs and Stars Task

#### Accuracy score (*d*′ prime)

**Figure 2 fig2:**
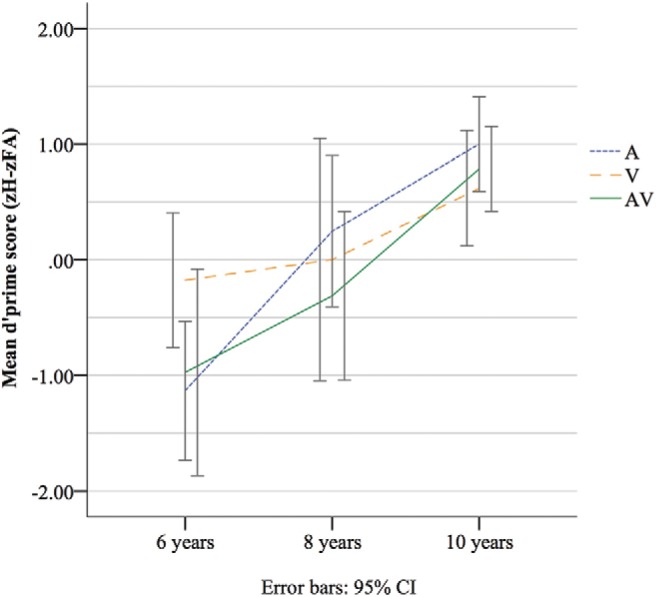
Mean accuracy score (*d*′prime) on MALT (zHit rate − zFalse Alarm rate) in each age group across CT conditions.

To examine target-detection accuracy on the MALT, z-scores for hit rates (H = correct hits/80 target trials) and FA rates (FA = false alarms/120 nontarget trials) were calculated. A *d*′prime [*d*′ = z(H) − z(FA)] measure of sensitivity was then calculated and mean values were analyzed across groups (see Figure 2). Results of a univariate ANOVA with two fixed factors of age and CT condition found a significant main effect of age, *F*(2, 171) = 17.71, *p* < .001, partial η^2^ = .17, but not of CT condition (*F* < 1); showing no effect of CT modality on accuracy performance across groups. No significant Age × Condition interaction was found, *F*(4, 171) = 1.47, *p* = .213. Bonferroni-corrected post hoc tests found that 6-year-olds were significantly less accurate than 8- and 10-year-olds (*p* = .013, and *p* < .001, respectively). In addition, 8-year-olds had significantly lower accuracy scores than 10-year-olds (*p* = .006), demonstrating improvement in task accuracy with age.[Fig-anchor fig2]

#### Errors on concurrent task (CT)

**Figure 3 fig3:**
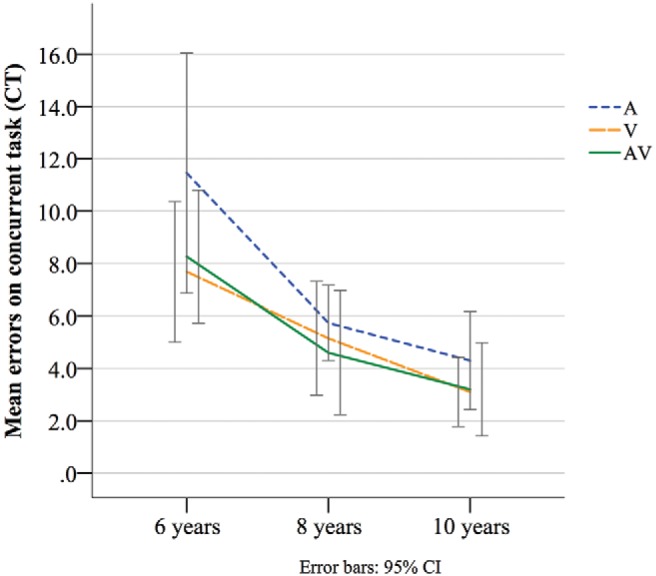
Mean number of errors on concurrent task (CT) in each age group across CT conditions.

Mean number of errors made on the concurrent task (counting stars [V], dings [A], or dinging stars [AV]) were calculated for each age group and across CT conditions (see Figure 3). Given that counting was started anew for each block, error scores for each block were calculated as an absolute deviation score from correct number of stars, dings, or dinging stars (deviation either higher or lower). Total error score for each participant was the sum of deviation errors across blocks.[Fig-anchor fig3]

Results of a univariate ANOVA found a significant main effect of age on number of errors on concurrent task, *F*(2, 170) = 16.57, *p* < .001, partial η^2^ = .16, but no effect of condition, *F*(2, 170) = 2.25, *p* = .11. Bonferroni-corrected post hoc tests found significantly more errors in 6-year-olds than 8- and 10-year-olds (*p* < .001 for both), but not between 8- and 10-year-olds (*p* = .11).

With the inclusion of DSB score (as a proxy for working memory) as a covariate in this analysis, the pattern of results remained, with main effect of age, *F*(2, 169) = 19.23, *p* < .001, partial η^2^ = .16, and no effect of condition, *F*(2, 169) = 1.85, *p* = .16. No significant Age × Condition interaction was found (*F*<1).

### Incidental Category Learning Task

#### Total correct

**Figure 4 fig4:**
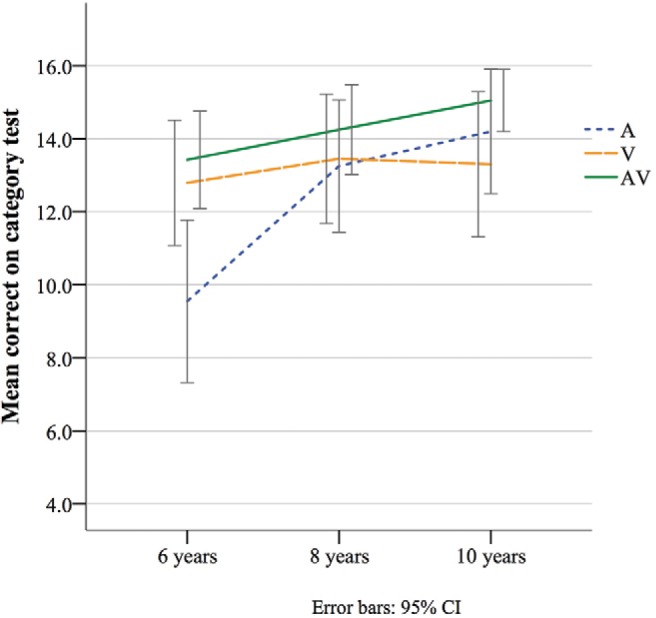
Mean correct on category task in each age group across CT conditions.

Mean number correct (out of 16 trials) on the incidental category learning task were calculated for each group (see Figure 4). Results of a univariate ANOVA found a significant effect of age, *F*(2, 171) = 6.38, *p* = .002, partial η^2^ = .07, an effect driven by 6-year-olds scoring significantly fewer correct than 8- and 10-year-olds (Bonferroni-corrected: *p* = .01 and .001, respectively). A significant effect of CT condition was also identified, *F*(2, 171) = 4.19, *p* = .02, partial η^2^ = .05, with fewer correct following the auditory-only (A) than the audiovisual (AV) concurrent task conditions (*p* = .004), with no significant differences between any other conditions (Bonferroni-corrected: *p* > .05 for all). Although no significant Age × CT Condition interaction was identified, *F*(4, 171) = 2.05, *p* = .09, partial η^2^ = .05, planned comparisons were conducted to examine the effects of condition within each group separately. Results found a significant effect of CT Condition only in the 6-year-old group, *F*(2, 60) = 6.43, *p* = .003, partial η^2^ = .18. Bonferroni-corrected comparisons showed that this was due to poorer category test performance in the 6-year-olds who were given an auditory concurrent task compared to either a visual-only (*p* = .043) or an audiovisual (*p* = .003) concurrent task. No significant difference in category learning was identified between visual and audiovisual CT (*p* = 1).[Fig-anchor fig4]

#### Performance from chance

Performance from chance on the category identification task was calculated for each age group and condition. Results of two-tailed *t* tests with a test value of eight showed that participants scored significantly above chance in each group and for all conditions (*p* < .001 for all), except for 6-year-olds in the auditory-only CT condition, *t*(21) = 1.45, *p = .16*.

#### Relationships between CT, incidental category learning, and DSB

In an investigation of whether performance on the CT task (number of errors on counting concurrent dings/stars/dinging stars) was related to incidental category learning (number correct on category test), Pearson correlations found no significant relationship in any CT condition (*p* > .05 for all). In addition, no significant relationships were found between CT and DSB *T* scores in any CT condition (*p* > .05 for all). An examination of relationships between DSB and category learning performance also found no significant relationships in any CT condition (*p* > .05 for all).

#### Age and performance on incidental category learning task

To examine changes in performance on the categorization task with age, we carried out Pearson’s correlations, which revealed a significant positive relationship between age and total correct in the incidental category learning task in the auditory-only condition (*r* = .43, *p = .001*), and a trend in the AV condition (*r* = .25, *p = .06*) but not in the visual condition (*p = .95*).

## Discussion

This study investigated the effect of concurrent task modality on incidental category learning, across middle childhood. Performance in the youngest children, aged 6 years, on an incidental category learning task was significantly poorer when engaged in an auditory CT than with a visual or audiovisual CT; a finding not seen in the older children. Our findings are, therefore, indicative of age-related changes in susceptibility to distraction when a concurrent task requires attention only to the auditory modality, in this case counting “dings.” Paradoxically, this was not found to be the case when a visual element was presented simultaneously with the concurrent auditory information in the bimodal (audiovisual) CT condition in any age group. Here, the concurrent visual information seemed to somehow compensate for the negative effect of auditory information described above. Similarly, in the youngest group, when CT stimuli consisted only of unimodal visual information, notably less detriment to incidental category learning was found than with an auditory CT.

These results are suggestive of cross-modal differences in the effects of cognitive load across childhood. In particular, engagement in an additional auditory task is particularly detrimental to performance on a multisensory learning task. In contrast, the findings indicate that the addition of visual information may serve to focus an individual’s attention on the visual features of the learning task, resulting in better incidental learning of category information. This may have been by the automatic capturing of attention to the most salient or familiar stimuli (Christie & Klein, 1995), or by filtering out the auditory information (Macdonald & Lavie, 2011).

On the main MALT task, age-related differences in performance were found, in line with expected developmental changes in attention, inhibition, and performance on continuous performance tasks (Levy, 1980; Lin, Hsiao, & Chen, 1999) and concurrent load tasks (Irwin-Chase & Burns, 2000; Karatekin, 2004). Importantly, however, no differences in task elements such as MALT accuracy score or concurrent task performance were found across CT conditions, indicating that incidental category learning in the current study was not a reflection of differences in performance on the main task. Instead, our findings suggest that children’s attentional resources are differentially affected depending on the modality of the concurrent task stimuli.

It was initially proposed that there might be heightened cognitive load from concurrent tasks that required attention to bimodal properties (in the present case, audiovisual information), and that this would result in poorer learning outcomes on a central task than with a unimodal concurrent task. This was anticipated particularly in younger children for whom the ability to integrate bimodal information into a unitary percept may not be fully mature (e.g., Gori et al., 2008; Nardini et al., 2015), thus resulting in a greater level of information to be processed in two sensory modalities. In contrast, however, when a concurrent task included bimodal information, this resulted in better performance on an incidental learning task than a concurrent task that only included auditory information. This is in line with the *intersensory redundancy hypothesis* (Bahrick et al., 2004), that would suggest amodal cross-modal stimuli might be processed more efficiently and without increasing cognitive load.

Prima facie, is seems unclear from the current results whether allocating attention to a concurrent auditory task is *detrimental* to performance or whether, to the contrary, it is the exposure to a visual or audiovisual concurrent task that leads to an *improvement* in category learning per se. That is, the presentation of multisensory information may lead to more efficient processing of the concurrent task stimuli, thus reducing cognitive resources required to complete both tasks. However, this would also result in a difference in incidental learning between visual and audiovisual CT conditions, a difference not found in the current study, suggesting that this cannot fully explain the results.

To further address this point, performance in each group would have to be compared with performance on the primary learning task without a concurrent task. In the study by Broadbent et al. (2017), a comparable attention task with audiovisual stimuli resulted in performance in incidental category learning that was above chance in 6-year-olds; a finding of multisensory facilitation on learning that is also supported by other studies (e.g., Jordan & Baker, 2011). Although a direct comparison of data from the two studies was not possible due to task modifications, at-chance performance in the present study using an audiovisual task with an auditory CT suggests that attention to the concurrent auditory information *did* result in a detriment to learning in 6-year-olds.

The effects of multisensory distractors are reduced in conditions of high perceptual load in young children, but not in adults (Matusz et al., 2015). Although it is unclear the extent to which the MALT involves a low or high perceptual load, the current study differs from the task used by Matusz et al. (2015)in that the peripheral multisensory information was presented as an intermittent concurrent task to which participants were asked to attend, rather than serving as distractor items to be ignored. Our findings therefore provide a unique insight into the role of unisensory and multisensory information that is secondary to a primary learning task when the stimuli from both are to be processed.

Van der Burg, Olivers, and Theeuwes (2012) suggest one mechanistic explanation for how attentional focus may narrow with an increase in task demands in children. According to these authors, multisensory information may shield children from distraction. This could only be corroborated in future studies with the addition of adult participants, to examine whether mature cognitive systems would show multisensory distractor effects.

In the study by Matusz et al. (2015), at levels of low cognitive load, 6-year-olds responded significantly faster in the presence of auditory distractors than with visual-only and audiovisual distractors, suggesting that unimodal auditory information is not processed as readily as visual information in this age group. This finding is somewhat in contrast with our results, which suggest additional auditory information may be processed to a greater extent, or at least impacts to a greater extent on incidental learning than visual-only or audiovisual concurrent stimuli. However, our findings may also reflect developmental changes in a modality processing dominance. Our results could, therefore, be considered as consistent with an auditory processing dominance in younger children (Sloutsky & Napolitano, 2003), with auditory information in our task seemingly more salient and therefore more likely to be processed than concurrent visual stimuli. Others have also reported age-related differences in auditory processing (e.g., Nava & Pavani, 2013). However, unlike in the current study, learning was not evaluated, nor were there any secondary or “distractor” stimuli to attend to. In addition, in modality dominance studies, cross-modal cues are typically presented in synchrony and so it remains unclear whether the presentation of auditory information in a concurrent task that occurred after the visual input on a primary task would pull attention in a comparative way.

If modality dominance were a causal factor in our results, it might also be expected that participants engaged in an audiovisual concurrent task would perform at a similar level to those in the auditory-only condition, with the auditory information serving as an attention-grabber, particularly in 6-year-olds. In fact, engaging in a multisensory concurrent task resulted in high learning performance in all groups. Moreover, no correlation was identified between performance on the CT (ability to count CT stimuli) and incidental learning in any condition, indicating that performance in category learning was not necessarily a reflection of greater processing or enumeration of auditory concurrent task stimuli, but a result of processing information in different modalities across two separate tasks.

The ability to switch flexibly across modalities is a skill undergoing development in the early years, with a gradual maturation of voluntary control over shifts of attentional set during childhood (Colombo, 2001; Garon, Bryson, & Smith, 2008; Jones, Rothbart, & Posner, 2003). This may go some way to explaining age-related differences when faced with a concurrent task modality that differs from the central task modality. On an audiovisual task such as the MALT, where the integration of multisensory information may not be optimal in younger children (e.g., Barutchu et al., 2009; Gori et al., 2008; Nardini et al., 2015), it may be that young children in particular were attending primarily to one element of the stimuli. For instance, focusing predominantly on the visual aspect of the MALT stimuli would have resulted in less distraction when faced with a concurrent visual or audiovisual task than with a concurrent auditory task, which would require the individual to flexibly shift across modalities. That said, this can only be assumed from the data as it is plausible that participants across conditions could rely exclusively on one modality for both the CT and MALT aspects of the task. Alternatively, it could be argued that the youngest children were actually focusing more on the auditory aspects of the MALT targets in order to acquire categorical information. In this situation, CT stimuli in the same modality might have heightened cognitive load and resulted in greater distraction. However, this is unlikely to be the case, given that when young children are only presented with unimodal auditory cues that are informative to categorical learning, they perform more poorly than with visual-only or bimodal audiovisual information (Broadbent et al., 2017). On this task, Broadbent and colleagues compared category learning performance (without an additional concurrent load task) in 5- to 10-year-olds when category membership was defined either by visual-only, auditory-only or multisensory information. Here, participants demonstrated superior performance following exposure to bimodal cues, and with the poorest performance seen in the youngest children who were presented with auditory-only category information.

Findings from dual-task studies suggest that focusing attention to stimuli in one modality can encumber performance in a different modality (e.g., see Strayer & Johnston, 2001). However, if it can be assumed that participants were switching of attention between modalities on the two tasks in the present study, this was not seen to negatively impact the detection of visual events in the MALT. That is, the concurrent task modality did not impede the ability to identify the target (frog), as shown on the accuracy measure on the MALT, but only the subsequent incidental learning of categorical features from multisensory targets. It could be suggested therefore that shifts of attentional set across modalities may be more deleterious to incidental learning of category information than to young children’s ability to respond to a target. Alternatively, these findings may merely reflect the different effects of CT stimuli on a complex categorical learning task compared with a relatively simpler detection task. Of note is that although age-related differences in performance were seen following an auditory concurrent task, only a trend toward a significant Age × Condition interaction was found on the incidental learning task, and so planned comparisons to examine these developmental changes should be considered with some vigilance. This said, a strong positive correlation between age and incidental learning in the auditory CT condition group is further support of developmental changes in the effects of an auditory concurrent task on learning.

In children aged 9 and 11 years, during the detection of auditory targets, auditory and audiovisual distractors were found to result in higher error rates than with visual distractors and with visual or audiovisual targets (Downing et al., 2015). This suggests that cross-modal distractors may have differential effects on the processing of multisensory targets. In contrast to our study, Downing, Barutchu, and Crewther (2015) presented visual distractors in temporal synchrony with targets, but required a shift in spatial attention to the periphery. Visual concurrent task stimuli in the present study, however, were presented in the same spatial location as targets. CT stimuli were also not presented in temporal synchrony with the stimuli on the primary task and so may not have served as a cross-modal “distractor” in the same way. This highlights an important distinction between the current study and previous work examining the role of unisensory and multisensory distractors on multisensory processing. In essence, the processing of multisensory stimuli for purposes of learning may be differentially effected by engaging in cross-modal concurrent tasks compared with in the presence of extraneous and temporally synchronous distractors, particularly in younger children.

In summary, in children below 8 years of age, engagement in a concurrent auditory task modulated incidental learning performance to a greater extent than with visual or audiovisual stimuli. This suggests that intermittent auditory information on a concurrent task may have a different effect on performance than visual or bimodal stimuli. The results of the present study extend developmental research on cross-modal processing and provide novel insight into the differential role of unisensory and multisensory concurrent tasks on incidental category learning in children. Developmental improvements in multisensory integration and ability to switch flexibly between modalities are likely to underpin changes in the role of concurrent task modality on learning across the primary school years. Such findings have implications for designing educational environments and learning tasks and are indicative of changes with age in the ability to process multisensory information on two concurrent tasks, particularly when switching between modalities is required.
